# Development of a novel, high-affinity ssDNA trypsin inhibitor

**DOI:** 10.1080/14756366.2019.1569648

**Published:** 2019-02-06

**Authors:** Stanislaw Malicki, Miroslaw Ksiazek, Pawel Majewski, Aleksandra Pecak, Piotr Mydel, Przemyslaw Grudnik, Grzegorz Dubin

**Affiliations:** a Malopolska Centre of Biotechnology, Jagiellonian University, Krakow, Poland;; b Department of Microbiology, Faculty of Biochemistry, Biophysics and Biotechnology, Jagiellonian University, Krakow, Poland;; c Department of Oral Immunology and Infectious Diseases, University of Louisville School of Dentistry, Kentucky, USA;; d Department of Clinical Science, Broegelmann Research Laboratory, University of Bergen, Bergen, Norway

**Keywords:** Aptamer, ssDNA, trypsin, protease inhibitor

## Abstract

Inhibitors of serine proteases are not only extremely useful in the basic research but are also applied extensively in clinical settings. Using Systematic Evolution of Ligands by Exponential Enrichment (SELEX) approach we developed a family of novel, single-stranded DNA aptamers capable of specific trypsin inhibition. Our most potent candidate (T24) and its short version (T59) were thoroughly characterised in terms of efficacy. T24 and T59 efficiently inhibited bovine trypsin with *K_i_* of 176 nM and 475 nM, respectively. Interestingly, in contrast to the majority of known trypsin inhibitors, the selected aptamers have superior specificity and did not interact with porcine trypsin or any human proteases tested. These included plasmin and thrombin characterised by trypsin-like substrate specificity. Our results demonstrate that SELEX may be successfully employed in the development of potent and specific DNA based protease inhibitors.

## Introduction

The design and development of small molecule inhibitors of proteases have proved an extremely daunting task due to selectivity problems. Trypsin has been extensively characterised as a type serine protease and a convenient model to investigate enzyme inhibition. Multiple inhibitors have been identified and their binding was thoroughly characterised. A large number of small molecule inhibitors have been developed which bind at the substrate binding cleft mimicking interactions of the substrate while in a majority of cases irreversibly modify the catalytic residues. Most of those inhibitors suffer from selectivity issues. A number of protein and peptide inhibitors have also been identified. These also bind at the active site preventing substrate access but avoid hydrolysis. While certain protein inhibitors are highly selective, in a majority of cases they are less suitable for medical applications. Only a very small number of exosite protease inhibitors have been described^1^^,^^2^.

Lack of specificity when targeting catalytic site is a recurrent topic and major challenge leading to the lack of specificity and potential life-threatening toxic effects. An interesting alternative is provided by oligonucleotide aptamers – molecules with single-stranded DNA or RNA scaffold, that bind target proteins with unique selectivity and high affinity, largely alleviating a significant number of problems associated small molecule inhibitors. Thanks to their exceptional properties (e.g. straightforward chemical modification, lack of toxicity, the possibility of *in vitro* synthesis), aptamers constitute an excellent tool for both, basic research and development of therapeutic strategies (e.g. target validation, imaging, etc.). Most importantly, aptamers have great potential for clinical use.

Several RNA aptamers capable of inhibiting proteolytic activity have been reported^3–7^. In this study, we describe the development and characterisation of a nano-molar single-stranded DNA aptameric inhibitor of bovine trypsin characterised by exceptional selectivity.

## Methods

All reagents and proteins, if not mentioned otherwise, were purchased from Sigma-Aldrich (Darmstadt, Germany).

### *In vitro* selection

Single-stranded DNA library (N50), composed of 50 nucleotides random region flanked by fixed primer binding sequences: 5′-CATGCTTCCCCAGGGAGATG-N_50_-GAGGAACATGCGTCGCAAAC-3′, was synthesised at 0.2 μM scale and HPLC purified (IBA, Germany). The N50 library was selected against Mag-Trypsin, commercially available bovine trypsin immobilised on magnetic beads (Clontech Laboratories, Inc. CA, USA) using SELEX protocol^8^ with modifications. Briefly, 0.3 µl of beads (3 μl in the initial cycle) were mixed with denatured (5 min at 92 °C) ssDNA pool from previous cycle of selection (N50 library in the initial cycle). After 30 min incubation at room temperature (RT) with gentle agitation, the beads were washed three times with selection buffer: PBS (phosphate-buffered saline pH 7.4) supplemented with 5 mM MgCl_2_, 10 mM KCl and 0.01% Tween20. Magnetic particle concentrator rack was used for washing. Beads were re-suspended in dH_2_O, the enriched ssDNA pool was recovered under denaturing conditions (92 °C for 5 min) and amplified in PCR. 400 μl of PCR mix containing 1 μM primers (unmodified ss50_For: 5′-CATGCTTCCCCAGGGAGATG-3′ and 5'-phosphorylated ss50_Rev: 5′-GTTTGCGACGCATGTTCCTC-3′), 0.5 mM dNTPs, 2.5 mM MgCl_2_, 1.25U/100 μl of *Taq* polymerase (Thermo Scientific) was prepared and mixed with 3–0.3 μl of beads with immobilised protein and bound aptamers. The PCR was performed for 30 cycles, consisting of denaturation at 95 °C for 2 min, annealing at 53 °C for 30 s, and extension at 72 °C for 30 s followed by final extension at 72 °C for 5 min. PCR products were extracted with phenol-chloroform-isoamyl alcohol mixture (Sigma-Aldrich, Germany) and precipitated with isopropanol overnight at −20 °C. After centrifugation, DNA pellets were washed twice with 70% ethanol, dried, dissolved in 100 μl of dH_2_O and subjected to λ exonuclease (ThermoScientific) digestion of phosphorylated strand to retrieve the unmodified strand (single-stranded DNA pool). Digestion was performed for 1.5 h at 37 °C, with gentle shaking, in 500 μl mixture containing 100 U of λ exonuclease. Digestion products (ssDNA) were extracted with phenol-chloroform-isoamyl alcohol mixture, precipitated and dissolved in 100 μl of dH_2_O, ssDNA samples were stored at −20 °C before next selection cycle. In order to eliminate non-specific binding of oligonucleotides, yeast tRNA (Invitrogen) and BSA (Bioshop, Canada) were added as competitors during incubation of ssDNA pool with immobilised trypsin (negative selection against empty beads was not conducted).

After ten initial selection cycles, unspecific PCR products became persistent. To remove unwanted products samples were electrophoresed in 10% polyacrylamide gels with 7 M urea in 0.5× TBE buffer for 90 min at 90 V at 4 °C. The band corresponding to 90 nucleotide long fragments were cut, repeatedly frozen and thawed for water extraction. Purified fragments were PCR amplified. DNA was extracted from with phenol-chloroform-isoamyl alcohol mixture and precipitated with isopropanol and then subjected to λ exonuclease digestion as described above. After 15th round of selection, the aptamers were cloned into a plasmid, amplified and sequenced (see Supplementary material – Cloning and sequencing). Analysis of obtained sequences was performed with T-Coffee (multiple sequence alignment) programme.

### ELISA binding assay

Binding of selected aptamers to bovine trypsin was evaluated by ELISA. 96-well microtiter plate (Nunc, Rochester, NY, USA) was coated with 100 μl of bovine trypsin (10 µg/ml in PBS) for 1 h at RT. All further steps were performed at RT. Unbound trypsin was removed by washing with PBS-T (PBS with 0.05% Tween20). Non-specific binding sites were blocked with 1% BSA in PBS-T for 1 h. Excess of BSA was removed by washing. Serial dilutions of biotinylated aptamers ranging from 0 to 4 µM in selection buffer (100 μl) were added to the wells and incubated for 1 h. To determine non-specific binding, control sequence (5′-biotin-CGTACGGTCGACGCTAGC-(ACTG)_12_A-CACGTGGAGCTCGGATCC-3′) was used. Unbound biotin-labeled aptamers were removed by extensive washing with selection buffer. 100 μl of horse-radish-peroxidase HRP-conjugated streptavidin (1:200 dilution; R&D Systems) was added to the wells. After 20 min incubation, unbound HRP-conjugated streptavidin was removed by washing and 100 µl of HRP reagent substrate (R&D Systems, MN, USA) was added. After 5–10 min, when a clearly visible blue colour developed, the reaction was stopped by the addition of 50 μl of 1 M H_2_SO_4_. The absorbance at 450 nm and 540 nm (correction for optical imperfections of the plates) was determined using a PowerWaveXmicroplate reader (BioTek).

### Immobilisation tests

To confirm the ability of selected aptamers to bind trypsin, immobilisation assay was used. 5'-biotin-labeled, HPLC purified ssDNA aptamers (4 μM; IBA, Germany) were incubated in selection buffer at RT for 30 min with 50 μl of Streptavidin M-280 Dynabeads (Invitrogen). The beads were washed three times with selection buffer in order to remove unbound aptamers and then incubated in selection buffer with bovine trypsin (16 μM in a total volume of 50 μl) for 20 min at RT with gentle agitation. The excess of trypsin was removed by washing with selection buffer. The beads were re-suspended in 20 μl SDS-PAGE loading buffer. The proteins were denatured (95 °C for 5 min) and resolved in 10% SDS-polyacrylamide gel. The gels were stained with Coomassie Brilliant Blue R-250.

### Binding kinetics analysis

The affinity of T24 aptamer for bovine trypsin was determined using Surface Plasmon Resonance (for details see Supplementary material – methods).

### Aptamer shortening

The sequence of the best-selected aptamer, T24, was divided into 10 nt long segments and nine mutational variants lacking one of these segments were obtained by chemical synthesis (Figure 3). Each aptamer mutant was analysed for its ability to inhibit bovine trypsin to determine regions within the aptamer sequence responsible for trypsin inhibition.

### Trypsin inhibition assay

Aptamers (0–10 µM) were incubated with trypsin (0.5 µM) and residual activity of the enzyme was determined using synthetic chromogenic substrate Nα-Benzoyl-L-arginine 4-nitroanilide hydrochloride (BAPNA). Activity was determined in the trypsin aptamer assay buffer (TAAB): 0.1 M Tris, 150 mM NaCl, 5 mM CaCl_2,_ 10 mM KCl, 5 mM MgCl_2,_ 0.02% Tween 20, pH 7.5 (for details see Supplementary material – methods).

### Determination of the inhibition mode and K_i_

Trypsin was active site titrated with 4-nitrophenyl 4-guanidinobenzoate^9^. Steady-state inhibition constant (*K_i_*) was determined as described previously^10^ employing model for mixed inhibition (for details see Supplementary material – methods).

### Screening of aptamer specificity

Bovine and porcine trypsin (200 nM), human plasmin (40 nM), human thrombin (20 nM), human neutrophil elastase (20 nM), human cathepsin G (40 nM), and porcine pancreatic elastase (20 nM) were preincubated with 50 molar excess of T24 and T59 aptamers in TAAB for 30 min at 37 °C. Relevant substrates were added: MeoSuc-AAPV-pNA (500 μM) for elastases, Suc-AAPF-pNA (500 μM) for cathepsin G, BAPNA (1.5 mM) for trypsins, Boc-QAR-AMC (100 μM) for thrombin and plasmin. Residual activity was monitored for 30 min at 37 °C using a SpectraMAX microplate reader (Molecular Devices, Sunnyvale, CA) for pNA substrates (absorbance at 410 nm) and SpectraMax Gmini XS (MolecularDevices) for Boc-Val-Pro-Arg-AMC (excitation: 380 nm, emission: 460 nm).

## Results

### Aptamer selection

Aptamers with affinity to bovine trypsin were selected from a random pool using SELEX methodology. After 15th round of selection, the pool was cloned into a plasmid and sequenced. Homologies were analysed among obtained sequences to identify six prevalent aptamer families: T3, T6, T21, T22, T24, and T35 (Table 1).

**Table 1.** Aptamers sequences.

Designation	Sequences
T3	CATGCTTCCCCAGGGAGATGACGCACATGTGGTTTTTAGACGGGCGTCCTGTGATGGGAGCGGGGTTAGGGAGGAACATGCGTCGCAAAC
T6	CATGCTTCCCCAGGGAGATGTCGGCGGGATATCGTGGCGGTTTGTTGGAGTTAGGGCATCTTCGGACTGGGAGGAACATGCGTCGCAAAC
T21	CATGCTTCCCCAGGGAGATGGGTGTGGGGTTAGGTCACTTTATGGTTGGTTCCATTGTGTCAGGGAGCCGGGAGGAACATGCGTCGCAAAC
T22	CATGCTTCCCCAGGGAGATGGGTGTGAGGTTAGGAAATCTGTGTTGAACACTGCTTTCAGGGGGCTTGGGAGGAACATGCGTCGCAAAC
T24	CATGCTTCCCCAGGGAGATGGGTGTGAGGTTAGTTTGCGGGAACTAGAATTCCTGGCAAAGGGGGGCCTGGAGGAACATGCGTCGCAAAC
T35	CATGCTTCCCCAGGGAGATGTGGCATAACCATCTCCGATAATATGGGTCACAGCTGGGCGGAAGGAGCTCGAGGAACATGCGTCGCAAAC
T59	CATGCTTCCCCAGGGAGATGGGTGTGAGGTTAGTTTGCGGTCCTGGCAAAGGGGGGCCTGGAGGAACATG
T159	CAGGGAGATGGGTGTGAGGTTAGTTTGCGGTCCTGGCAAAGGGGGGCCTGGAGGAACATG

### Analysis of binding capacity of selected aptamers

**Figure 1.**
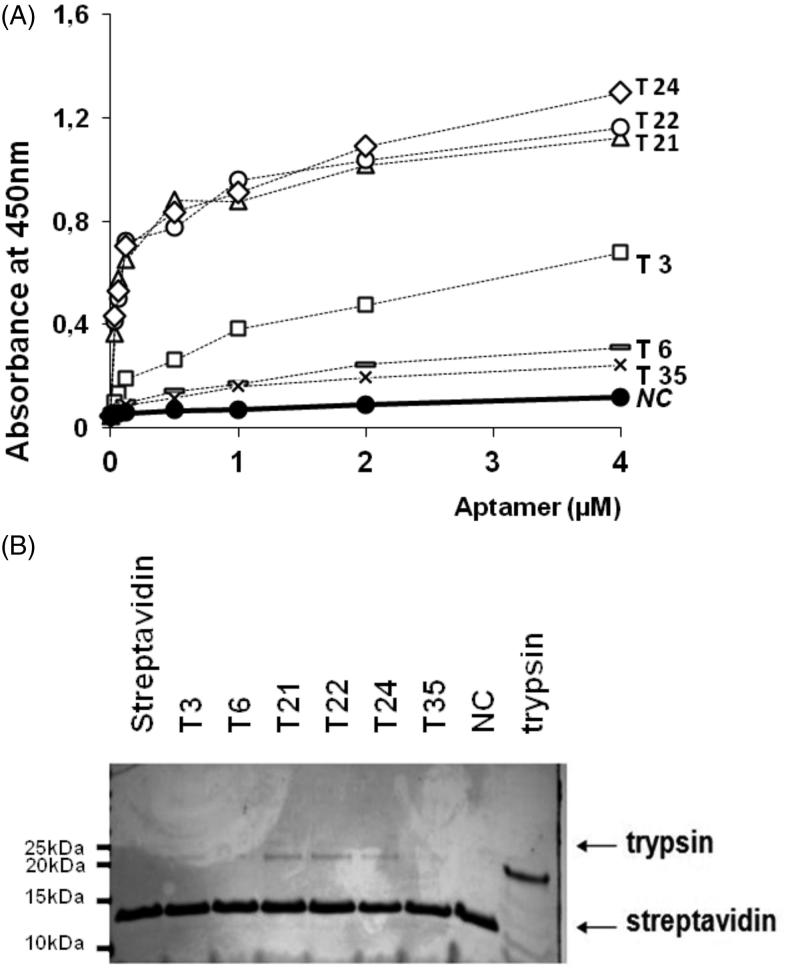
Interaction of aptamers with bovine trypsin. (A). ELISA. Biotinylated aptamers at indicated concentrations were incubated with immobilised trypsin and detected with HRP-streptavidin. NC - negative control. (B) Biotinylated aptamers were immobilised on magnetic beads. Beads were contacted with bovine trypsin and retained fraction was analyzed by SDS-PAGE after thorough washing.

The binding capacity of identified families towards bovine trypsin was analysed by ELISA. Our results showed that aptamers annotated as T21, T22, and T24 were characterised by highest and almost identical properties. T3 have only around 50% of the binding capacity of the previous three aptamers. Both T6 and T35 showed very low binding on the level of a non-specific sequence serving as a negative control (NC) (Figure 1(A)).

The results of the ELISA assay were verified in an alternative test where the interaction of trypsin with biotinylated aptamers immobilised on streptavidin conjugated to magnetic beads was tested. In line with previous results, T21, T22, and T24 were most efficient in retaining trypsin (Figure 1(B)), with T3 aptamer showing significantly weaker band intensity corresponding directly lower to trypsin affinity. Aptamers T6 and T35 did not show any visible binding remaining on the level of negative control.

### Trypsin inhibition

**Figure 2.**
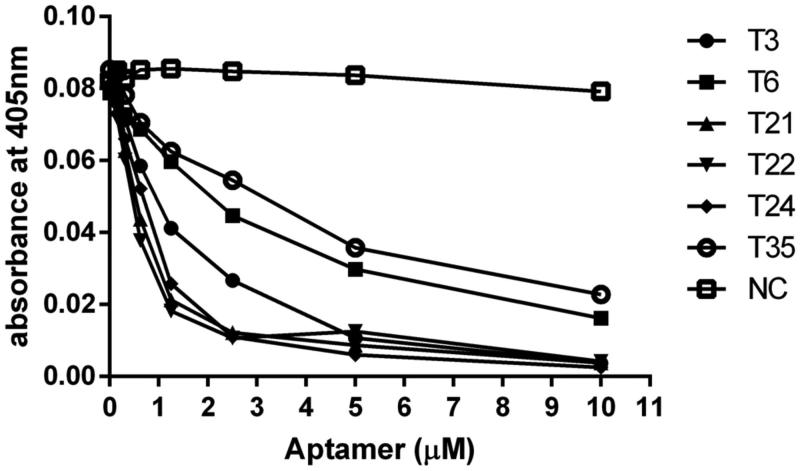
Inhibition of trypsin by aptamers. Aptamers at indicated concentrations were pre-incubated with bovine trypsin. Residual trypsin activity was determined using chromogenic substrate (L-BAPNA) by monitoring absorbance at 405 nm.

Although our previous experiments showed efficient binding of T21, T22, T24, and T3 to bovine trypsin, that might not directly correlate with enzyme inhibition. In order to verify the ability of the selected aptamers not only to bind but also to inhibit trypsin, residual activity of the enzyme was determined after pre-incubation with aptamers of interest (Figure 2). We found that the inhibitory efficacy of tested aptamers correlated closely with their binding capacity. T21, T22, T24, and T3 were the most efficient inhibitors whereas T6 and T35 were significantly less effective in our assay. Of note, although T6 and T35 have demonstrated in the previous experiments very weak interaction with the target molecule (on the level of negative control) they still provided significant inhibition of the target enzyme. This could be explained by the limitations of the binding assay that is not able to detect very weak interactions (compounds characterised by high K_d_). These aptamers were however not considered further due to poor inhibition. Of comparably behaving T21, T22 and T24, the latter was chosen for further analysis. The affinity of T24 aptamer towards trypsin has been characterised using Surface Plasmon Resonance demonstrating k_on_=1.03 × 10^6^ M^−1^s^−1^, k_off_=3.15 × 10^−3^ s^−1^, and the Kd of = 3.06 × 10^−9^ M (Supplementary Figure 2).

### Shortening of T24 aptamer

**Figure 3.**
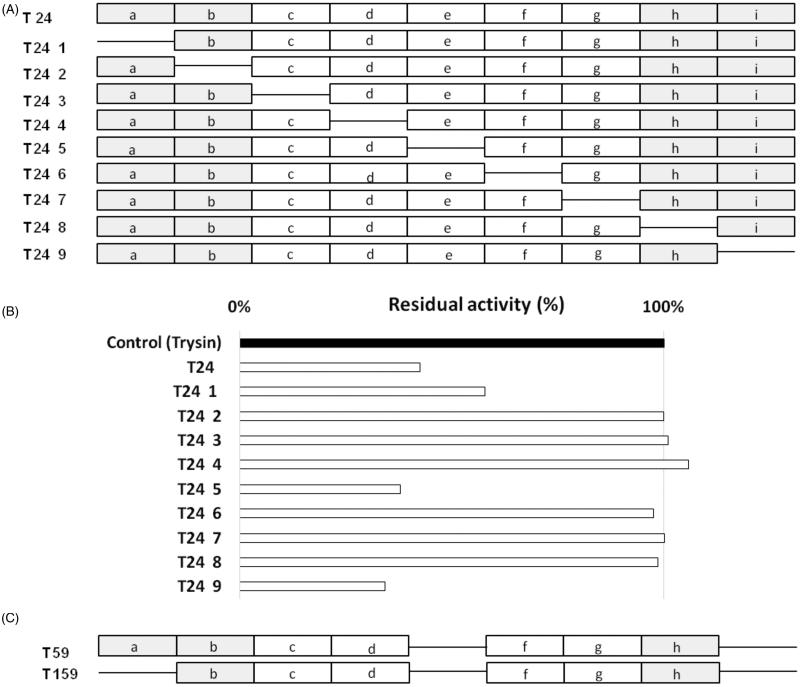
Identification of minimal sequence responsible for inhibitory capacity of T24. (A) Schematic representation of probing scheme – nine new aptamers were obtained each devoid of depicted region. Each region (a–i) represents 10 nucleotides. Primer interaction sites are shaded grey. (B) The inhibitory activity of aptamers depicted in panel A against bovine trypsin. Enzyme was pre-incubated with tested aptamer and residual activity was determined using L-BAPNA. (C) Schematic representation of two resulting versions of shortened T24 aptamer.

Primary aptamers identified using the SELEX approach originated from the relatively long initial library. In general, not all nucleic acids of the aptamers are necessary for the binding affinity between the aptamers and the targets. To determine the minimal sequence of T24 aptamer necessary for binding and inhibition of trypsin, the T24 was divided into 10 nucleotide long segments, to narrow down the sequence region responsible for the target binding (Figure 3). Segments (a, b, h and i) originated from the conserved flanking sequence of the library (primer attachment sites) while segments (c–g) encompassed the variable region of the library. All variants were obtained where each particular segment was missing and their inhibitory activity towards trypsin was screened. Removal of any of the segments (b–d, f–h) resulted in complete loss of inhibition demonstrating the importance of these regions for trypsin interaction or aptamer structure. Conversely, removal of segments a, e or i had no effect on inhibition demonstrating that these regions of T24 aptamer are not involved either in inhibition or aptamer structure stabilisation (Figure 3).

**Figure 4.**
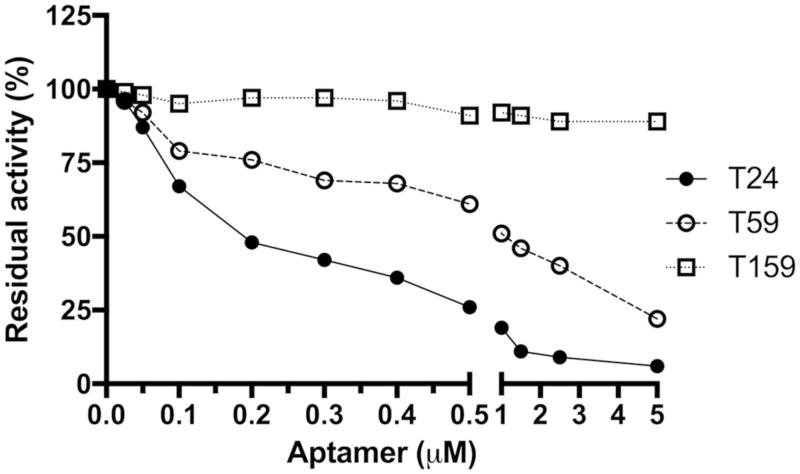
Dose-dependent inhibition of trypsin by aptamers: T24, T59, and T159. Trypsin was preincubated in the presence of indicated concentrations of aptamers before the residual activity was determined using chromogenic substrate (L-BAPNA).

Based on the above findings two shortened versions of T24, namely T59 (segments e and i removed) and T159 (segments a, e and i removed) were obtained (Table 1 and Figure 3(C)) and further characterised. Interestingly, T59 preserved the inhibitory capacity of parent aptamer T24 while T159 lost the inactivity (Figure 4).

### Determination of inhibition constant

To further compare the inhibitory potency of T24 and T59 and potentially determine the mode of inhibition kinetic analysis of inhibition was performed. The inhibition curve followed the Michaelis–Menten kinetics and was dependent on both substrate and aptamer concentration (Supplementary Figure 1). Extrapolations of the simple linear regression model fitted to the experimental points crossed away from any of the axes on Lineweaver–Burk plot (Supplementary Figure 1). This data clearly shows that the inhibition of trypsin by tested aptamers is reversible and best described by a mixed-model of enzyme inhibition. The steady-state inhibition constant (*K_i_*) was determined as a parameter most suitable for describing the potency of reversible inhibitors. T24 and T59 inhibited trypsin with *K_i_*of 176 ± 16 and 475 ± 48 nM, respectively.

### Analysis of inhibition specificity

**Figure 5.**
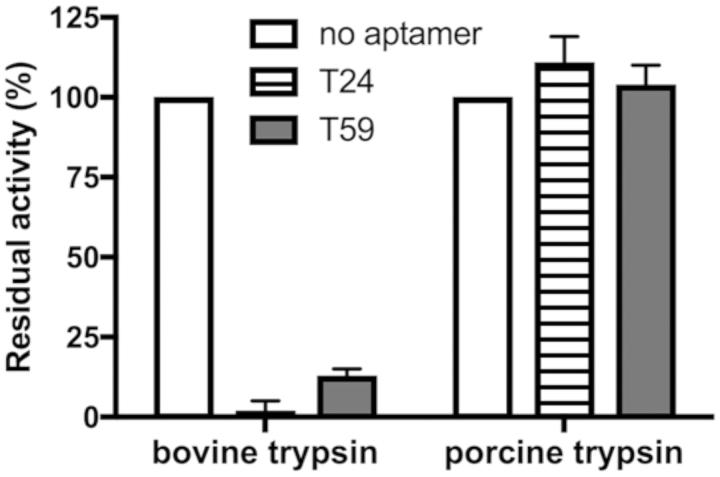
Aptamers selected towards bovine trypsin do not inhibit porcine homolog. Bovine and porcine trypsin were pre-incubated with T24 and T59. Residual proteolytic activity was determined using chromogenic substrate (L-BAPNA) by monitoring absorbance at 405 nm.

**Figure 6.**
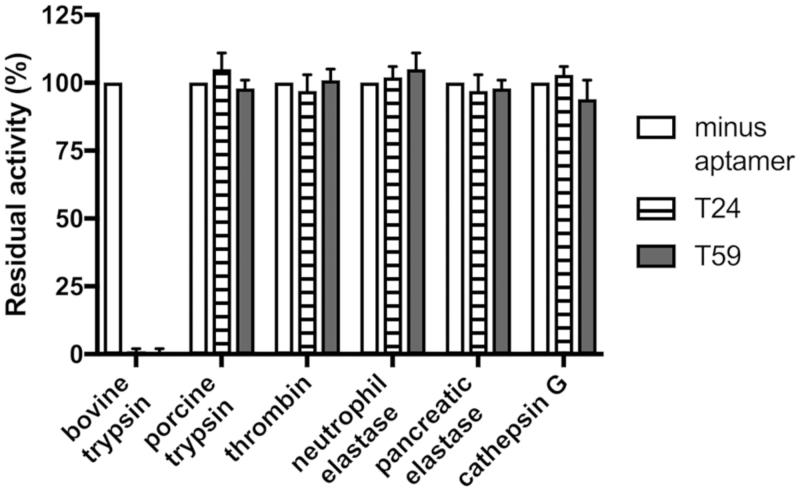
Trypsin aptamer specificity. Mammalian proteases: porcine pancreatic elastase, human cathepsin G, neutrophil elastase, plasmin, and thrombin were pre-incubated with T24 and T59. Residual proteolytic activity was determined using substrates relevant for each protease (see Materials and Methods).

To determine the specificity of interaction between the T24 and T59 aptamers and the target enzyme inhibition of a closely related porcine trypsin as well as commercially available mammalian proteases were tested. Presence of T24 or T59 had no impact on the activity of porcine pancreatic elastase, human cathepsin G, neutrophil elastase, plasmin and thrombin (Figure 5 and Figure 6) demonstrating that both our candidates are not only efficient but also highly selective. Such high specificity is a unique feature of aptamers as compared to small molecule inhibitors and also greater than that of many protein/peptide inhibitors.

## Discussion

Trypsin is not only a model enzyme in investigations of proteolysis but also a highly relevant clinical target. A large number of natural protein/peptide inhibitors and small molecule inhibitors of trypsin have been reported, with the former being usually characterised by better specificity.

A popular low molecular weight inhibitor nafamostat mesilate (FUT-175), a synthetic serine protease inhibitor often used in basic medical research, inhibits trypsin with *K_i_* of 15 nM but presents low specificity inhibiting a number of proteases pivotal in coagulation cascade^11^. Gabexate mesylate, another synthetic protease inhibitor, shown to be effective in treating patients with sepsis-associated disseminated intravascular coagulation, also exhibits significant cross-reactivity inhibiting human tryptase (*Ki* = 3.4 nM), bovine tryptase (180 nM) and trypsin (2 μM)^12^. Developed by us aptamers T24 and T59 inhibited trypsin with K_i_s of 176 and 475 nM, respectively. Affinity values of our aptamers, however, higher than the synthetic inhibitors described above have an undisputed advantage of high specificity. Neither T24 nor T59 had any effect on tested elastases, cathepsin G, plasmin and thrombin. Not even the activity of porcine trypsin was affected. That level of specificity ensures selectivity even at higher concentrations compensating lower affinity and potentially eliminating undesired side effects. That level of specificity is comparable with protein inhibitors^13^, which unlike aptamers are highly complex in production, expensive and hard to scale up. Small molecules are usually more promiscuous as compared to aptamers or proteins/peptides. For example, gabexate despite targeted at human tryptase (*Ki* = 3.4 nM), also inhibits thrombin (500 nM), plasmin (1.5 μM), factor Xa (2 μM) and trypsin (2 μM). Nafamostat inhibits not only trypsin (*Ki* = 15 nM), but also thrombin (84 nM), C1r (14 nM), C1s (38 nM), tryptase (95 pM), plasmin and kallikrein^11^. Broad specificity is a common problem limiting the use of small molecules not only in clinical setting but also in the laboratory.

Low specificity of small-molecule inhibitors is directly related to their mechanism of action. They interact directly with amino acids within the catalytic cleft of target enzymes, which are usually not specific for particular protease, but rather the entire protease family characterised by a similar mechanism of action/substrate specificity^14^. Due to large molecular weight, aptamers avoid such localised interactions or supplement them with additional interactions with the surface residues. Surface residues, in turn, are not involved in catalysis or substrate binding and, therefore, are highly variable and characteristic for particular enzymes. Such a conclusion is supported by available crystal structures. Analysis of twelve available structures of aptamer-ligand complexes deposited in Protein Data Bank (PDB) brings a general observation as to a molecular mechanism of aptamer binding – all aptamers characterised in this regard interact only with the well-exposed surface residues and charged residues are preferred. A similar mechanism is expected for T24 and T59 which most likely bind at the surface of trypsin blocking the access of the substrate to catalytic cleft by steric hindrance. This proposed mode of interaction would explain why our aptamers inhibit bovine, but not porcine trypsin despite comparable substrate specificity of these enzymes and 82% sequence identity (note that the differences are found mostly in surface exposed regions).

Analysis of Lineweaver–Burk plots and binding kinetics corroborates the hypothesis on aptamer binding. T24 and T59 inhibit trypsin in a mixed-model mechanism. The equation relevant to this model contains an α parameter (Supplementary methods), which is related to the mode of inhibition. When α = 1 the inhibitor does not alter the binding of the substrate and this situation is very similar to non-competitive inhibition. When α is large, inhibitor interaction prevents substrate binding and the model is similar to that of competitive inhibition. Small α signifies enhancement of substrate binding by the inhibitor (similar to uncompetitive model). T59 and T24 are characterised by α of 6.6 and 6.4 × 10^16^, respectively. This suggests that both aptamers inhibit trypsin through the mechanism very close to competitive inhibition.

A number of aptamers inhibiting the proteolytic activity of proteases have been reported (Supplementary Table 1). These molecules wary from 71 to 169 nucleotides and inhibit target proteases with *K_i_* ranging from 2.1 nM to 2.5 µM. The Kis of T24 and T59 (176 and 475 nM, respectively) are not differs in this regard. Interestingly, early all proteases targeting aptamers are characterised by RNA scaffold. The only reported DNA based inhibitor of protein C reached only 30% reduction of maximal enzyme activity^15^. RNA is considered better suited for designing of inhibitors because of the presence of a 2′-OH group and non-Watson-Crick base pairing allowing more diverse and complex three-dimensional (3D) structures of RNA oligonucleotides^16^^,^^17^. However, DNA is more stable than RNA and our study provides an important “proof-of-concept” that potent and highly specific protease inhibitors can be created using DNA scaffold.

In conclusion, we successfully created ssDNA aptamers targeted at bovine trypsin which inhibit the enzyme with nano-molar affinity and high selectivity. High selectivity is especially interesting considering the difficulty of achieving selectivity when targeting protease active sites leading to the toxic effects by the majority of known small molecular weight inhibitors. Our results demonstrate that SELEX is a suitable method for identification of highly selective and potent inhibitors of human proteases and offer the promise of providing a novel generation of therapeutic entities with a variety of applications.

## Supplementary Material

Supplemental Material
