# Supplementary material for: Development of a novel, high-affinity ssDNA trypsin inhibitor
Source: J Enzyme Inhib Med Chem. 2019 Feb 6;34(1):638–43. doi: 10.1080/14756366.2019.1569648 (PMC6366424; doi:10.1080/14756366.2019.1569648)
Supplement: Supplemental Material [file IENZ_A_1569648_SM1345.pdf]

Supplementary materials

Methods

Cloning and sequencing

PCR after the last selection cycle was carried out using unmodified primers: ss50_For and ss50_Rev. Products were ligated into the pTZ57R/T vector by TA cloning (InsTAclone PCR Cloning Kit; Fermentas). The resulting constructs were transformed into *E.coli* DH5α. Several dozens of colonies were tested for the presence of desired inserts by colony PCR with M13 primers (M13fwd: 5'-GTAAAACGACGGCCAGT-3', M13rev: 5'- CAGGAAACAGCTATGAC-3'). Plasmids from positive clones were isolated using mini prep kit (Plasmid DNA Kit, Bio Basic Inc.) and sequenced with M13 primers (Genomed, Poland).

Binding kinetics analysis

The SPR (surface plasmon resonance) was used to characterize T 24 aptamer interaction with bovine trypsin. Biotinylated aptamers were diluted to a concentration of 5 nM in HBS-P buffer pH 7.4 (GE Healthcare) containing 5 mM Mg^{2+}, boiled for 10 min at 95° C and cooled on ice for 10 min. 700 resonance units (RU) of a biotinylated T24 and biotinylated negative control aptamer were immobilized on the surface of a SA chip (GE Healthcare) according to the manufacturer's protocol. The binding kinetics was analysed in trypsin aptamer assay buffer (TAAB): 0.1 M Tris, 150 mM NaCl, 5 mM $CaCl_2$, 10 mM KCl, 5 mM $MgCl_2$, 0.02% Tween 20, pH 7.5 with Biacore X100 (GE Healthcare). A single 20-sec pulse of 2 M KCl was used for chip regeneration in multi-cycle kinetics. The binding data were fitted to the Langmuir 1:1 binding model using Biacore X100 Evaluation software.

Trypsin inhibition by full length aptamers

The inhibitory potential of full length aptamers against trypsin was tested using synthetic substrate: Nα-Benzoyl-L-arginine 4-nitroanilide hydrochloride (BAPNA). 1 µM bovine trypsin was incubated with increasing concentrations of aptamers (0 – 10µM) in selection buffer for 20 min at room temperature. BAPNA (in the same buffer) was added (final trypsin concentration 0,5 µM; final substrate concentration 1,5mM; total reaction volume 100µl) and the reaction was monitored at 405 nm using microplate reader at 37˚C for 1 hour.

Analogous experiments were performed using Nα-benzoyl-L-isoleucyl-L-glutamyl-glycyl-L-arginine-4-nitroanilide and the same reaction conditions.

Trypsin inhibition by T24 mutants

Bovine trypsin was active site titrated with 4-nitrophenyl 4-guanidinobenzoate [1] in trypsin aptamer assay buffer (TAAB): 0.1 M Tris, 150 mM NaCl, 5 mM $CaCl_2$, 10 mM KCl, 5 mM $MgCl_2$, 0.02% Tween 20, pH 7.5. Trypsin at concentration of 100 nM was mixed with increasing concentrations of aptamers in a microtitration plate to yield molar ratios of enzyme: inhibitor ranging from 0 to 50. After 15 min of incubation at 37°C, 100 µl of chromogenic substrate L-BAPNA solution was added to yield a final concentration of 1.5 mM, and enzymatic hydrolysis of substrate was monitored for 30 min at 37°C at 405 nm using SpectraMAX microplate reader. Residual activity was plotted as a function of aptamer concentration.

Determination of the inhibition mode and equimolar inhibition constant

Active site titrated trypsin (200 nM) was incubated at 37°C in TAAB in the presence of increasing concentrations of aptamers (0 to 600 nM) in a 96-well plate. After 15 min, the fluorogenic substrate L-BAPNA was added at several concentrations (0 to 1.5 mM) and the residual activities were recorded for 30 min at 37°C at 405nm using microplate reader. The type of enzyme inhibition was determined graphically using the Lineweaver–Burk plot according to the Eq. (1):

$$\frac{1}{V_0} = \frac{K_m + [S]}{V_{max} \times [S]} = \frac{K_m}{V_{max}} \times \frac{1}{[S]} + \frac{1}{V_{max}} \quad (1),$$

where V_0 is the initial reaction velocity, V_{max} the maximum reaction velocity, K_m the Michaelis-Menten constant (determined by nonlinear regression using GraphPad Prism), [S] the substrate concentration and K_i inhibition constant.

The inhibition constant K_i was determined by a curve fitting using Graph Pad Prism software (La Jolla, USA) and the macro for mixed inhibition and Eq. (2):

$$V_0 = \frac{V_{max} \times [S]}{K_m \times \left(1 + \frac{[I]}{K_i}\right) + [S] \times \left(1 + \frac{[I]}{\alpha \times K_i}\right)} \quad (2),$$

where α is a parameter which corresponds to the mechanism of inhibition.

Supplementary Literature

1. Chase, T., Jr., and Shaw, E. (1967) p-Nitrophenyl-p_-guanidinobenzoate HCl: a new active site titrant for trypsin. Biochem Biophys Res Commun. 1967 Nov 30;29(4):508-14.

2. Duclair S, Gautam A1, Ellington A2, Prasad VR1.High-affinity RNA Aptamers Against the HIV-1 Protease Inhibit Both In Vitro Protease Activity and Late Events of Viral Replication.MolTher Nucleic Acids. 2015 Feb 17;4:e228.
3. Lupold SE, Hicke BJ, Lin Y, Coffey DS. Identification and characterization of nuclease-stabilized RNA molecules that bind human prostate cancer cells via the prostate-specific membrane antigen. Cancer Res. 2002 Jul 15;62(14):4029-33.
4. Takeno H, Yamamoto S, Tanaka T, Sakano Y, Kikuchi Y. Selection of an RNA molecule that specifically inhibits the protease activity of subtilisin. J Biochem. 1999 Jun;125(6):1115-9.
5. Gal SW1, Amontov S, Urvil PT, Vishnuvardhan D, Nishikawa F, Kumar PK, Nishikawa S. Selection of a RNA aptamer that binds to human activated protein C and inhibits its protease function. Eur J Biochem. 1998 Mar 15;252(3):553-62.
6. Fukuda K, Vishnuvardhan D, Sekiya S, Hwang J, Kakiuchi N, Taira K, Shimotohno K, Kumar PK, Nishikawa S. Isolation and characterization of RNA aptamers specific for the hepatitis C virus nonstructural protein 3 protease. Eur J Biochem. 2000 Jun;267(12):3685-94.

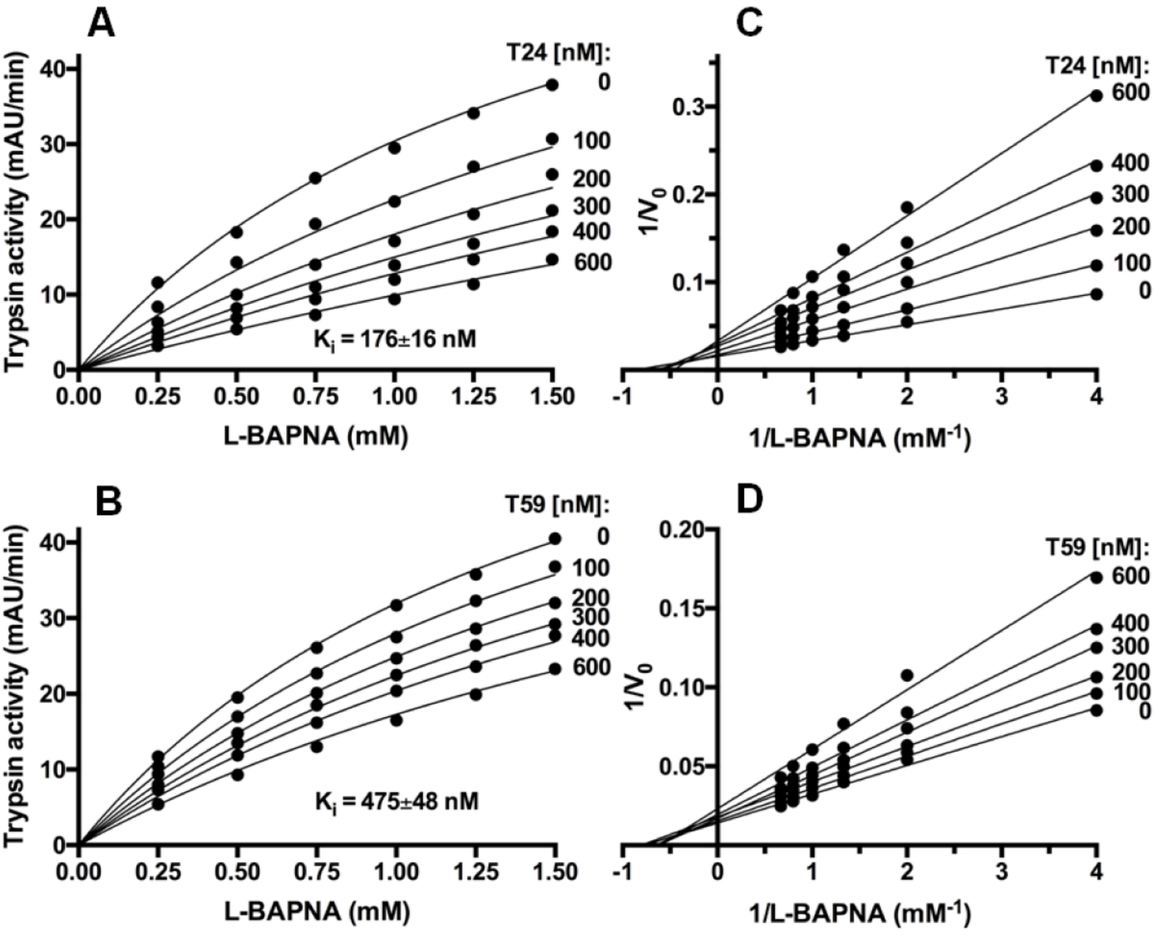

Supplementary figure 1. Steady-state kinetics of trypsin inhibition by aptamers. Trypsin was pre-incubated in the presence of increasing concentrations of aptamers. After pre-incubation, activity was determined at several different concentrations of L-BAPNA. (A and B) Michaelis-Menten plot of the rate of substrate hydrolysis (Ki was determined by nonlinear regression using GraphPad Prism). (C and D) Lineweaver–Burk plot of reciprocal initial reaction velocity (V_0) versus reciprocal substrate concentration [S]. All extrapolated fits cross at single point and away of either axis signifying mixed mechanism of inhibition.

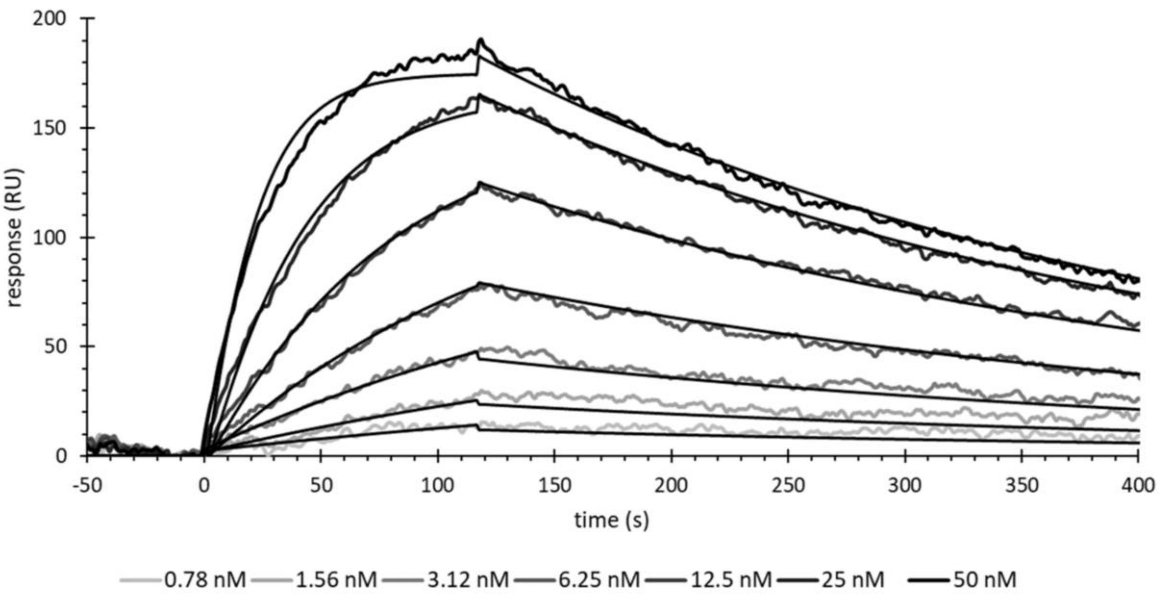

Supplementary figure 2. SPR analysis of T24 interaction with trypsin. Representative sensograms are shown ($K_D = 3.06 \times 10^{-9}$ M, $k_{on} = 1.03 \times 10^{6}\ M^{-1}s^{-1}$, $k_{off} = 3.15 \times 10^{-3}\ s^{-1}$).

Table 1. Known aptameric inhibitors of proteases.

**Molecular target**	**Type (DNA/RNA); name; length (nt)**	**K_i [nM] (mode of inhibition, if determined)**
HIV-1 aspartyl protease (PR)	RNA; PR10.13; 96	138 (noncompetitive) [2]
	RNA; PR10 1-8A; 96	31,5 nM (noncompetitive) [2]
Prostate-specific membrane antigen (PSMA)	RNA; xPSM-A9; 71	2.1 (noncompetitive) [3]
	RNA; xPSM-A10; 71	11.9 (competitive) [3]
Subtilisin	RNA; RNA-1; 82	2500 (competitive) [4]
Human activated protein C (APC)	RNA ; APC-167; 169	83 (noncompetitive) [5]
	RNA; APC-99; 99	137 (noncompetitive) [5]
The viral encoded non-structural protein 3 (NS3)	RNA; G9-I; 74	100 (noncompetitive) [6]
